# Novel technique of flexible endoscopic therapy of Zenker’s diverticulum using a pulsed Holmium laser—a retrospective single-center analysis

**DOI:** 10.1007/s00464-022-09775-w

**Published:** 2022-11-30

**Authors:** Franz Singhartinger, Antonia Gantschnigg, Iris Mühlbacher, Andrej Wagner, Oliver Koch, Klaus Emmanuel, Josef Holzinger

**Affiliations:** 1 Department for Surgery, University Hospital Salzburg, Paracelsus Medical University, Salzburg, Austria; 2 Department for Internal Medicine I, University Hospital Salzburg, Paracelsus Medical University, Salzburg, Austria

**Keywords:** Zenker, Diverticulotomy, Dysphagia, Holmium laser

## Abstract

**Background:**

In recent years, flexible endoscopic therapy of Zenker’s diverticulum seems to become the standard approach. The aim of this study was to assess the safety, efficacy and short-term outcome of flexible endoscopic diverticulotomy of Zenker’s diverticulum with a pulsed Holmium laser (PHL).

**Patients and methods:**

All patients treated with endoscopic laser-diverticulotomy using a PHL between February 2013 and November 2021 at the University Hospital Salzburg were extracted from our prospectively maintained endoscopic database. Demographic data, size of Zenker’s diverticulum, procedure duration, complications, short-term outcome and rate of recurrence were evaluated.

**Results:**

In the study period, 45 procedures in 36 patients were performed. Mean depth of the Zenker diverticulum was 21 mm (10–60 mm), mean procedure time was 31 min (15–60 min), intraprocedural adverse events occurred in 2 out of 45 patients (5%) which were both managed endoscopically, post-procedural stenosis occurred in 1 patient (2%). In the follow-up examinations (mean follow-up after 6.4 months), 27 out of 36 patients (75%) were symptom-free, 6 patients (17%) reported an improvement of dysphagia. 3 patients (9%) suffered from persistent dysphagia. After initial symptom relief, a recurrent diverticulum occurred in 5 patients. Endoscopic re-intervention with PHL was done in these cases.

**Conclusions:**

Flexible endoscopic treatment of primary and recurrent Zenker’s diverticulum using a PHL is a promising, safe and effective treatment with, in our opinion, technical advantages in comparison to the CO_2_ laser. Further controlled prospective trials are needed.

**Supplementary Information:**

The online version contains supplementary material available at 10.1007/s00464-022-09775-w.

Esophageal diverticula are a rare finding. The most commonly known esophageal diverticulum, the Zenker’s diverticulum (ZD), is a false diverticulum of the pharyngoesophageal junction. It arises in the Killian’s triangle which consists of the two bellies of the inferior pharyngeal constrictor muscle, the cricopharyngeus and thyropharyngeus, being part of the upper esophageal sphincter [1]. Although ZD was first reported in 1769 by Ludlow [2], the anatomy of the upper esophageal sphincter is still a matter of debate [3].The suspected pathogenesis of the ZD is an impaired relaxation of the upper esophageal sphincter or uncoordinated cricopharyngeal muscle contraction, which causes a pulsion diverticulum in the preformed low resistance muscular area [4]. ZD occurs mainly in the 7th and 8th life decade and has a male predominance. The incidence is 2/100.000 persons/year [1, 4–6]. Main symptoms include halitosis, dyspnoea and regurgitation of solid food and fluids. Leading symptom is dysphagia.

Symptomatic ZD is an indication for interventional treatment. This can be open surgical diverticulectomy (OSD), rigid endoscopic diverticulotomy (RED) with a stapler or flexible endoscopic diverticulotomy (FED). In recent years, FED seems to become the standard procedure for ZD with similar outcomes and lower morbidity and mortality in comparison to OSD and RED [4, 7]. Different approaches of FED have been published. Direct cutting techniques with different endoscopic devices such as clutch cutter, hook knife or CO_2_ laser differ from techniques creating a submucosal space like the Z-POEM technique [8].

The aim of this retrospective trial is to evaluate the safety, efficacy and short-term-outcome of flexible endoscopic laser-diverticulotomy (FED-LD) using a novel technique with a pulsed Holmium laser (PHL).

## Patients and methods

All patients endoscopically treated by FED-LD from February 2013 until November 2021 in our high volume surgical endoscopy unit ( ~10.000 endoscopies p.a.) at a tertiary university hospital were included in this retrospective investigation. Retrospective data was extracted from our prospectively maintained endoscopic database.

Inclusion criteria were diagnosed ZD with the typical symptom of cervical dysphagia. Exclusion criteria were none.

Analyzed criteria were demographic data (age, sex), depth of ZD, procedure duration, intra- and postinterventional adverse events, improvement of dysphagia in follow-up and the necessity of re-intervention due to recurrent ZD.

Agreement by the ethics committee responsible for our institution was obtained (ethics committee of Salzburg, Austria, EK-Nr.: 1016/2022).

### Pre-interventional diagnostics

Patients were referred to our center either because of dysphagia or previously diagnosed ZD via gastroscopy and/or contrast-swallow x-ray.

Patients who recently underwent external gastroscopy were planned for intervention, patients without external gastroscopy underwent a diagnostic gastroscopy prior to the intervention. Pre-interventional contrast-swallow x-ray was not obligatory since we don’t recognize additional diagnostic value if typical dysphagia and an endoscopically visualized ZD is present. All patients were hospitalized for the intervention. Written informed consent was obtained from all patients.

### The pulsed Holmium Laser (PHL)

The PHL is used in modern medicine for different indications on soft tissue in the prostate and solid calculi in the kidney [9, 10]. Usage in refractory bile duct stones has been reported [11, 12]. PHL produces a radiation with a wavelength of 2140 nm, has a short distance of action and thereby reduces the risk of advertent injury to surrounding structures in comparison to the CO_2_-laser.

Furthermore, the PHL has the capability of working in environments such as air, saline and wet tissue in opposite to the CO_2_ laser, which is almost exclusively absorbed by water [13]. Of note, incidents of airway fire caused by CO_2_ laser have been reported [14, 15], the PHL seems to have no such risk features.

### Interventional technique

**Fig. 1:**
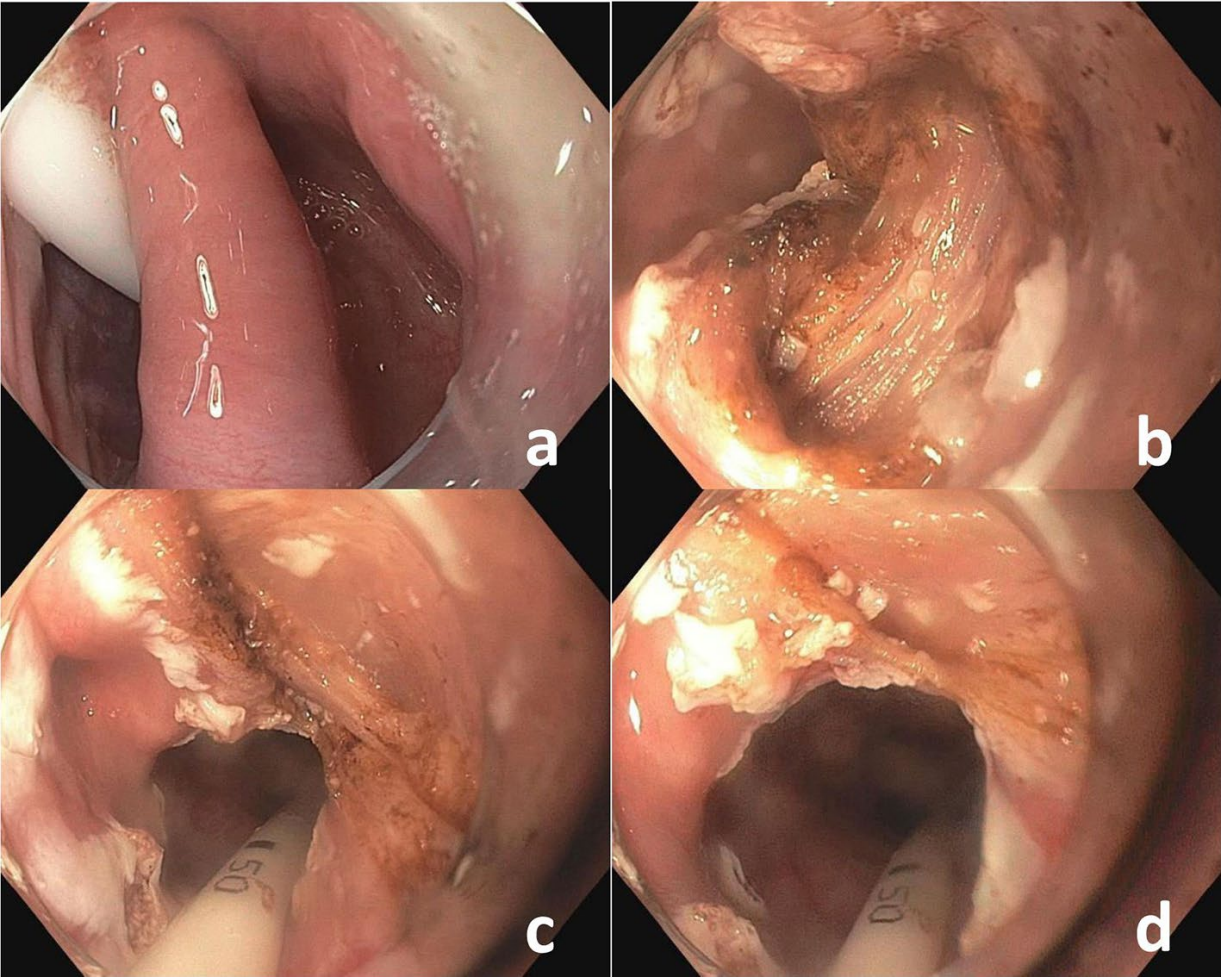
**a** Prior to intervention an oro-gastric tube is inserted; **b** visible muscle fibers after cutting the mucosa; **c** after subtotal cut of the cricopharyngeal muscle; **d** the cricopharyngeal muscle is completely divided

Diverticulotomy was done under continuous sedation with i.v.-administered Propofol 1% using standard gastroscopes (Olympus GIF HQ180 and HQ190, Olympus, Tokio, Japan) under carbondioxyde insufflation. A distal attachment cap (Olympus, Tokio, Japan) was used to better visualize the common wall between the diverticulum and the esophagus and to stabilize endoscope position. After placing a standard oro-gastric tube (Freka, CH 15, 100 cm, Fresenius Kabi, Bad Homburg, Germany) the mucosa of the common wall of the ZD was incised with the PHL (Lumenis Be Ltd., Yokneam, Israel). Subsequently the muscular wall of the ZD was cut through to the bottom of the diverticulum with the same laser. The distal margin of the incision was reached when the muscular wall of the ZD was completely divided and the muscular layer of the esophageal wall was reached (Video 1, Fig. 1). The oro-gastric tube was removed at the end of the procedure. On the first postoperative day a swallow attempt was carried out, and if swallowing was possible, the patients were kept on a soft diet for 3 days. The patients were discharged from the hospital 2 days after the procedure. Follow-up gastroscopy in an out-patient setting was planned 3 months after the intervention.

### Statistics

Only descriptive statistics were carried out due to the retrospective character of our investigation with no control cohort. Data was analyzed using mean values and standard distribution.

## Results

Between February 2013 and November 2021, 45 consecutive FED-LD in 36 patients took place in our surgical endoscopy unit. All interventions were done by four experienced endoscopists with at least 1000 endoscopic procedures per year. Further demographic data is provided in (Table 1). Mean depth of ZD, evaluated during endoscopy, was 21 mm (10–60 mm). Intraoperative adverse events were reported in 2 of 45 interventions (4%). In these cases a transmural defect appeared during the intervention which was closed by endoscopic clips and antibiotic treatment was startedAcknowledgements. No further treatment was necessary.

**Table 1:** Demographic data

Demographic data	
Patients	36 (female: 13; male: 23)
Procedures	45
Age	74 years (57–92)
Depth of diverticulum	21 mm (10–60 mm)
Duration of intervention	32 min (15–60 min)
Adverse events	2 (both treated endoscopically)
Technical success	45 (100%)
Clinical success at inital follow-up	
No dysphagia	27/36 (75%)
Improved dysphagia	6/36 (17%)
Persisting dysphagia at initial follow-up	3/36 (8%)
Delayed dysphagia	3/36 (8%)
Lost to follow-up	7/36 (19%)
Re-Intervention due to recurrence	10 procedures in 6 patients (1×4 re-interventions, 1×2 re-interventions, 4×1 re-intervention)

Median time until follow-up gastroscopy was 6.4 months (1.5–57).

In 7 patients no follow-up gastroscopy was carried out for different reasons (patient died from other disease, patient did not appear to follow-up gastroscopy).

Dysphagia completely dissolved in 27 out of 36 patients (75%), an improvement of dysphagia was reported by 6 patients (17%) and 3 patients (8%) suffered from persistent dysphagia at follow-up gastroscopy. 1 of these 3 patients suffered from dysphagia because of fibrotic stenosis weeks after the intervention which was successfully treated endoscopically with a single bouginage. The other 2 had recurrent ZD in a follow-up gastroscopy 3 months after FED-LD. In 5 patients (14%) delayed recurrence of dysphagia due to recurrent ZD after initial symptom relief appeared after 9, 12, 18 and 24 (2x) months, respectively. Recurrent ZD was again treated with FED-LD in all 5 patients.

Three of 5 patients reported complete symptom relief after the second treatment with FED-LD. One patient was treated 2 times with FED-LD until dissolvement of dysphagia. One patient was treated 4 times with FED-LD due to recurrent ZD. Finally OSD with stapled resection was carried out. Because of postoperative dehiscence of the esophageal wall, endoluminal vacuum therapy followed by endoluminal stenting was necessary.

Average duration of the intervention was 32 (15–60) minutes which improved over the years as an expression of a learning curve.

## Discussion

This is, to our knowledge, the first report about a patient cohort treated with FED-LD with a PHL. In the upper aerodigestive tract PHL has been used for treatment of benign tracheal stenosis with extremely low rate of adverse events [13]. In our study, the safety of FED-LD has been demonstrated with a low rate of intraoperative adverse events of 4% (2 cases) which were both managed endoscopically during the same intervention without any need for further interventional treatment. As a potential risk factor, one of these 2 cases was a recurrent ZD after RED and FED with argon plasma cautery. Published literature about OSD and FED have a high heterogeneity on the rate of adverse events ranging between 0 and 15% [16–18].

Recurrence rates are comparable or even lower than in other flexible endoscopic techniques [4, 7]. All but one recurrence appeared in the first 13 of 36 patients treated with FED-LD as an expression of a learning curve. This might be due to a restrained depth of diverticulotomy which is a natural observation in newly established interventional therapies. Indeed, a connection between depth of diverticulum, age or gender and recurrence rate could not be detected.

Comparison with a cohort treated with OSD is difficult because recent studies focus on FED alone. A study by Chang et al. from 2004 compared 24 cases with CO_2_ laser endoscopic diverticulotomy to 28 cases with open diverticulectomy with cricopharyngeal myotomy [19]. Although recurrence rates were higher in the endoscopic diverticulotomy group (21% vs. 0%) the duration of the intervention was significantly lower (47 vs. 170 min). This could be confirmed by another retrospective study by Koch et al. in 2011 [20]. Length of hospital stay was significantly lower in the endoscopic group in this investigation.

As far as we know now, it seems as if the endoscopic approach overall (with different techniques) has a less invasive character and therefore less post-surgical complications, reduced length of hospital stay and reduced costs but a higher recurrence rate.

As a limitation of our investigation, the follow-up period in our patient cohort is quite short (6.4 months; 1.5–57) and so late recurrences might not be taken into account.

A clinical success (improvement or dissolvement of dysphagia) was achieved in 30 out of 36 patients after the first treatment. Unfortunately, delayed recurrences (*n *= 5) appeared after 9, 12, 18 and 24 (2x) months. Re-interventions were safe and effective using the same technique. Only one patient required surgery due to a five time recurrence with a very large ZD.

The functioning and characteristics of the PHL are comparable with a Thulium laser. Data about the feasibility and safety of this method, compared with endoscopic stapled diverticulotomy, has recently been published [18]. This study showed that, although the endoscopically visible recurrence rates were higher in the FED-LD group (36.8%) in comparison to the RED (17.6%), quality of life was higher in the FED-LD group after a median follow-up-period of 16 months.

In our opinion the advantage of this method compared to stapled diverticulotomy and FED with other techniques like clutch cutter or hook knife is the clear visibility of the single muscle fibers of the cricopharyngeal muscle during the intervention as presented in (Video 1). Z-POEM provides clear visibility of target structures, but the depth of diverticulotomy may not be estimated as exactly as in the presented PHL technique. Furthermore Z-POEM has a noticeably longer median procedure time [17].

An advantage of the PHL in comparison to the CO_2_ laser is the short wavelength and thereby short penetration of the tissue resulting in a clean and sharp cut [13]. Therefore, a complete dissection of the common wall of the diverticulum without increasing the risk of transmural defects is possible.

A diverticuloscope, as recommended by other endoscopists [21] was not used in our interventions because the flexibility of movement is reduced and therefore the optimal placement of the laser beam is not possible. Recent data seems to confirm our approach [22].

In a review by Yuan et al. [16] with more than 6000 cases on endoscopic and open surgical treatment of ZD stated, that prospective trials on OSD versus FED are not present.

Beard et al. [23] describes the flexible endoscopic approach, with different devices (Argon cautery, hook knife, needle knife, triangle knife, bipolar or monopolar forceps) as the standard approach in modern medicine because of comparable recurrence rates and lower rates of adverse events in comparison to OSD.

Most studies and case series report about using general anesthesia and prophylactic antibiotics. We want to state, that none of our patients required general anesthesia, all interventions were done only in continuous sedation with Propofol. Prophylactic antibiotics were only used, when intraoperative transmural damage was detected (2 out of 45 cases, 4%). Therefore the cost effectiveness of FED-LD versus OSD is quite obvious. In our cost calculation the OSD would be about three times as expensive as FED-LD (2048.70 vs. 738.62 Euro). This calculation is not even regarding the longer inpatient stay after surgery.

The rate of persisting or recurrent dysphagia (17%) might be explained by the experimental character of the intervention in the beginning of our trial, and therefore insufficient myotomy. Nevertheless, FED with a PHL is an intervention especially tailored for older, comorbid patients. So the risk/benefit calculation of a minor intervention versus the surgical trauma with OSD for an old patient needs to be taken into account.

Limitations of our study are the lack of a score for dysphagia, no comparison with other methods, the short follow-up period and the heterogeneous patient cohort including patients after surgical or other endoscopic treatments (FED with APC) of ZD.

At last, FED-LD with a PHL seems to be a promising approach with low expense (except the laser itself), high effectiveness, low rate of adverse events and minimal distress for the patient. Nevertheless, prospective clinical investigations are needed.

## Supplementary Information

Below is the link to the electronic supplementary material.

Supplementary file1 (MP4 235023 KB)
